# Learning Mobile Manipulation through Deep Reinforcement Learning

**DOI:** 10.3390/s20030939

**Published:** 2020-02-10

**Authors:** Cong Wang, Qifeng Zhang, Qiyan Tian, Shuo Li, Xiaohui Wang, David Lane, Yvan Petillot, Sen Wang

**Affiliations:** 1 State Key Laboratory of Robotics, Shenyang Institute of Automation, Chinese Academy of Sciences, Shenyang 110016, China; wangcong2@sia.cn (C.W.); tianqiyan@sia.cn (Q.T.); shuoli@sia.cn (S.L.); wxh@sia.cn (X.W.); 2 Institutes for Robotics and Intelligent Manufacturing, Chinese Academy of Sciences, Shenyang 110016, China; 3 University of Chinese Academy of Sciences, Beijing 100049, China; 4 School of Engineering & Physical Sciences, Heriot-Watt University, Edinburgh EH14 4AS, UK; D.M.Lane@hw.ac.uk (D.L.); Y.R.Petillot@hw.ac.uk (Y.P.); s.wang@hw.ac.uk (S.W.)

**Keywords:** mobile manipulation, deep reinforcement learning, deep learning

## Abstract

Mobile manipulation has a broad range of applications in robotics. However, it is usually more challenging than fixed-base manipulation due to the complex coordination of a mobile base and a manipulator. Although recent works have demonstrated that deep reinforcement learning is a powerful technique for fixed-base manipulation tasks, most of them are not applicable to mobile manipulation. This paper investigates how to leverage deep reinforcement learning to tackle whole-body mobile manipulation tasks in unstructured environments using only on-board sensors. A novel mobile manipulation system which integrates the state-of-the-art deep reinforcement learning algorithms with visual perception is proposed. It has an efficient framework decoupling visual perception from the deep reinforcement learning control, which enables its generalization from simulation training to real-world testing. Extensive simulation and experiment results show that the proposed mobile manipulation system is able to grasp different types of objects autonomously in various simulation and real-world scenarios, verifying the effectiveness of the proposed mobile manipulation system.

## 1. Introduction

Robot manipulation, as one of the most fundamental and challenging research topics in robotics, has attracted significant interest in last decades. Based on the traditional dynamic control techniques, industrial robot manipulators can perform tasks repeatedly with high precision. However, most of the existing manipulation systems are fixed in structured environments with no or limited perception capability. Therefore, they are not adequate for a broad range of tasks and applications in practice, which requires reliable operation in unstructured and dynamic environments. Mobile manipulation is gradually in high demand.

Recently, deep reinforcement learning (DRL) has endowed robots with many new powerful capabilities, which were not possible several years ago, for example, general door opening [1], housework [2], learning agile walking for legged robotics [3]. The deep reinforcement learning method has become a new enabler for complex tasks, which are challenging to accomplish for the traditional methods.

Deep reinforcement learning has been widely adopted to learn deep visuomotor polices for fixed-base manipulation [4,5,6,7], achieving the state-of-the-art performance. However, mobile manipulation is much less investigated due to the high complexity and big challenge of coordinating a mobile base and a manipulator. It needs to produce coherent policies for both manipulation and mobility, being a holistic system that searches and interacts with a target using vision.

In this paper, we propose a novel mobile manipulation framework based on deep reinforcement learning. Since mobile manipulation mostly needs to operate in unstructured environments, an on-board RGB camera is introduced as a visual perception system. The framework is designed to decouple visual perception from the deep reinforcement learning based policy, enabling its direct generalization from simulation training to real-world testing. After extensive simulation evaluation, the trained mobile manipulation policy is tested on a real mobile manipulation robot to grasp various types of objects from random initial locations. To the best of our knowledge, this framework is the first deep reinforcement learning based mobile manipulation that is successfully deployed on a real-world robot.

The paper is organized as follows. In Section 2, related work on mobile manipulation is reviewed. Section 3 presents the proposed mobile manipulation system. In Section 4, simulation results of the mobile manipulation are described, followed by a real-world experiment in Section 5. Section 6 draws the conclusion of the paper.

## 2. Related Work

Robot manipulation is one of the main research topics in the robotics community, ranging from structured pick-and-place manipulation to unstructured field mobile manipulation. With the recent development of deep learning, there has been an increase in interest in reinforcement learning and robot control problems. This section reviews some related work in robot manipulation and reinforcement learning (RL).

### 2.1. Mobile Manipulation

Mobile manipulation has attracted significant interest in the community, including humanoid mobile robots, wheeled mobile robots and multi-legged mobile robots with one or two manipulators. Recently, some robotics challenges have also focused on mobile manipulation, such as DARPA Robotics Challenge [8] and the Mohamed Bin Zayed International Robotics Challenge (MBZIRC) [9]. These challenges are located in complex unstructured and dangerous environment such as disaster rescuing, and need the robots to perform a series of complex tasks combining perception, manipulation, localization and even multi-robot cooperation. The robots should have manipulation ability with a high-level requirement [10,11]. Houseroom is another common application situation, which is unstructured and hard for robots to do the housework. There are also some mobile manipulators designed for this task, such as PR2 [12], Human Support Robot (HSR) [13].

Efficiently controlling a mobile manipulator is an important yet open question, especially in unstructured dynamic environments. In Reference [14], the authors present a whole-body optimal control framework to jointly solve the problems of manipulation, and the optimization is performed using a Model Predictive Control (MPC) approach. The approach is tested in end-effector pose tracking and door opening tasks. In Reference [15], the authors develope object pose estimation using point cloud data and an improved iterative closest point algorithm method. With the pose input, the robot can select and adjust its pose by maximizing its manipulability based on some algorithms and criteria that the authors proposed. The autonomous mobile manipulator system is tested in the simulator and real robot. In Reference [16], the authors develop a supervised autonomous locomotion and manipulation robot for disaster response. Due to its high number of degrees of freedom, the authors propose supervised autonomy approaches to increase quality and speed of control while keeping the flexibility to solve unknown tasks.

### 2.2. Reinforcement Learning for Manipulation

With the recent development of deep learning, reinforcement learning has received more attention in robot control field. Using deep reinforcement learning, a large number of new complex robot applications have been achieved, such as playing cube [6], dexterous manipulation [17], learning to walk [18], learning agile and dynamic motor skills for legged robots [3], learning ambidextrous robot grasping [7]. In Reference [5], the authors propose a learning-based approach to hand-eye coordination for robotic grasping from monocular images. They train a large Convolution Neural Network (CNN) to predict the probability of successful grasps, using only monocular camera images independent of camera calibration or the current robot pose. Finally, the robot can successfully grasp novel objects. This experiment illustrates that learning-based approach has a great potential for robotic applications. In Reference [19], the authors propose a programming-by-demonstration approach to achieve catching different flight objects. They use a new methodology to find a feasible catching configuration in a probabilistic manner, which can enable a rapid adaptation of the arm motion. Later, this group also develops a dynamical system approach for softly catching a flying object [20]. They use a dynamic system-based control law to generate the appropriate reach and follow motion, propose a method to approximate the parameters of linear parameter varying (LPV) systems using Gaussian mixture models. In Reference [21], the authors proposed a system that can learn to throw arbitrary objects. They use an end-to-end formulation that jointly learns to infer control parameters for grasping and throwing motion primitives from visual observations through trail and error. The system is able to grasp and successfully throw arbitrary objects into boxes located outside its maximum reach range at 500+ mean picks per hour and generalizes to new objects and target locations.

There is also some research about reinforcement learning in mobile manipulation. In Reference [22], the authors propose a system that enables mobile manipulation robot to learn an action-related places through experience-based learning with the environment. The model is acquired through experience-based learning, which takes into account the robot hardware, control programs and interactions with the environment. In Reference [23], the authors propose HRL4IN, a novel Hierarchical RL architecture for interactive navigation tasks, applied on the mobile manipulation tasks, such as door opening, but not testing in real world. In Reference [24], the authors propose an approach to learn joint robot base and gripper action models using learning from demonstration method. They formulate a graph optimization problem that links observations and kinematic constraints. The test shows that the robot can learn how to open and drive through a door. In Reference [25], the authors use reinforcement learning strategy for a humanoid-like mobile manipulator. The strategy includes a high-level online redundancy resolution based on the neural-dynamic optimization algorithm in operational space and a low-level RL in joint space based on the dynamic movement primitives. The system can suppress the uncertain external perturbations. In Reference [26], the authors propose a one-shot visual appearance learning method for a mobile manipulator, which can robustly detect specific objects in a scene following an initial segmentation hint from a human user. They evaluate the algorithm performance under many different challenging conditions.

Compared with the traditional method and the learning-based method in mobile manipulation above, our method only uses the model-free reinforcement learning method to control a whole-body mobile manipulator, without any human teaching or demonstration. We have transferred the policy from the simulation to real robot in a typical mobile manipulation task successfully. What is more, it is easy to expand our framework to more complex tasks or different floating-based manipulators, such as flying robot or underwater robot.

## 3. Method

In order to solve complex mobile manipulation tasks, a mobile manipulator system usually consists of different subsystems, such as a mobile base, arm, gripper and vision system. In this paper, we consider the mobile manipulator as a whole-body controlled through deep reinforcement learning. Therefore, the learning-based mobile manipulation system proposed is composed of two parts: deep reinforcement learning control and visual perception.

### 3.1. System Overview

**Figure 1:**
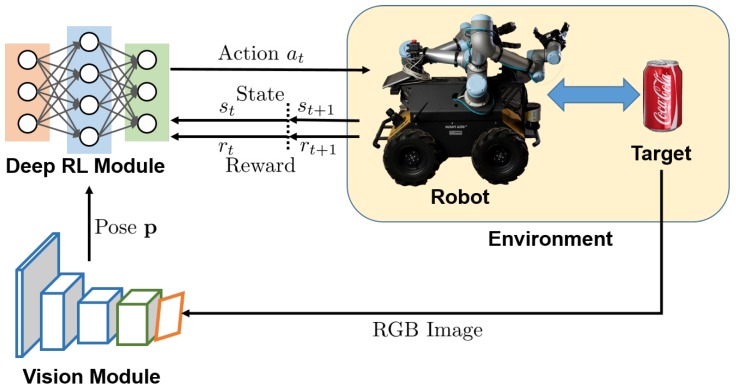
Learning-based mobile manipulation control framework. There are mainly two parts, deep reinforcement learning module and vision module. First, the vision module estimates the object 6-degrees of freedom (DoF) pose p from images captured by an on-board RGB stereo camera. Then, based on the object pose p and current robot state $s_{t}$, deep reinforcement learning module predicts an action $a_{t}$ for the robot to act. A new state $s_{t+1}$ and a reward $r_{t+1}$ are received after action.

Consider mobile manipulation as a standard reinforcement learning problem, in which an agent interacts with an environment to maximize the reward based on a policy. Following this paradigm, the deep reinforcement learning based mobile manipulation framework proposed in this paper is given in Figure 1. The deep reinforcement learning module obtains the target pose p and the current robot state $s_{t}$, predicts a control action $a_{t}$ for the mobile base and arm, and receives the new state $s_{t+1}$ and reward $r_{t+1}$.

### 3.2. Deep Reinforcement Learning

#### 3.2.1. Background

In the framework of reinforcement learning, the policy π(a|s) predicts an action a∈A based on a state s∈S and a reward r∈R is received after action. Specifically, at each control step, the agent observes the current state $s_{t}$ and samples an action $a_{t}$ from the policy π. Then the environment responds with a new state $s^{\prime }=s_{t+1}$ and a scalar reward $r_{t+1}$. The goal of a reinforcement learning problem is to learn the optimal parameters $\theta^{*}$ that maximize the expected return

(1) $$J\left(\theta \right)=E_{\tau ∼p_{\theta }\left(\tau \right)}\left[\sum_{t=0}^{T}\gamma^{t}r_{t}\right],$$

where $p_{\theta }\left(\tau \right)=p\left(s_{0}\right)\prod_{t=0}^{T-1}\left[\left(s_{t+1}|s_{t},a_{t}\right)\pi_{\theta }\left(a_{t}|s_{t}\right)\right]$ is the distribution over all possible state-action trajectories $\tau =(s_{0},a_{0},s_{1},\ldots ,a_{T-1},s_{T})$, $r_{t}$ is the reward received at time *t*, $\gamma^{t}\in \left[0,1\right]$ is the discount rate at time *t*. Policy gradient method is a common and effective method to solve this problem [27]. The policy gradient can be calculated as

(2) $$\nabla J\left(\theta \right)=E_{s_{t}∼d_{\theta }\left(s_{t}\right),a_{t}∼\pi_{\theta }\left(a_{t}|s_{t}\right)}\left[\nabla \log\left(\pi_{\theta }\left(a_{t}|s_{t}\right)\right)A_{t}\right],$$

where $d_{\theta }\left(s_{t}\right)$ is the state distribution under the policy $\pi_{\theta }$. $A_{t}$ is an advantage function

(3) $$A_{t}=R_{t}-V\left(s_{t}\right).$$

#### 3.2.2. Definition of States and Actions

The state and action spaces are key for the design of deep reinforcement learning algorithms for the mobile manipulation which has extra complexity to be generalized in practice compared with stationary manipulation. Therefore, the dynamic property needs to be well considered for the definition of the states and the actions. First, the mobile base state, i.e., its pose in the world, is represented in a relative local coordinate system instead of an absolute pose in a global frame. This brings in an extra advantage that the trained deep models are easy to generalize to a new configuration without fine tuning. Hence, the 6-DoF object pose can also be estimated in the local coordinate frame.

Second, the 6-DoF object pose is estimated by a dedicated vision perception system (more details in Section 3.3), instead of an end-to-end visuomotor learning scheme [4]. This is because the existing sim2real technique still has limited performance or requires significant computation for vision related reinforcement learning algorithms, due to the unrealistic visual rendering in the popular physical engines, like MuJoCo [28] and Bullet [29].

Therefore, our state space $s_{t}$ includes the position of the gripper w.r.t the robot base frame, the position of object w.r.t the gripper frame, the position of object w.r.t the robot base frame, the joint positions and velocities of the arm, as well as the gripper state. For the action space $a_{t}$, it is defined as three parts—the end-effector relative position control action $a_{arm}\left(\delta x,\delta y,\delta z\right)$ w.r.t the gripper frame, the robot base relative position control action $a_{base}\left(\delta x,\delta \theta \right)$ w.r.t the robot base frame, and the binary gripper action control $a_{grip}$.

#### 3.2.3. Policy Training

The Proximal Policy Optimization (PPO) algorithm is one of the most efficient model-free policy gradient methods and has achieved state-of-the-art performance in many reinforcement learning continuous control benchmarks [30]. It derives from the TRPO algorithm [31], but is easier to implement and also has better sample complexity. Therefore, PPO is employed in this work to train the defined reinforcement learning policy for the mobile manipulation.

PPO is an Actor-Critic type algorithm, so the value function is trained using multi-step returns with TD(λ) and the policy gradient is computed using the Generalized Advantage Estimator (GAE) [32]. The basic form of PPO is defined as:

(4) $$L_{\mathrm{PPO}}=\left[\min\left(\frac{\pi \left(a_{t}|s_{t}\right)}{\pi_{\mathrm{old}}\left(a_{t}|s_{t}\right)}{\hat{A}}_{t},\mathrm{clip}\left(\frac{\pi \left(a_{t}|s_{t}\right)}{\pi_{\mathrm{old}}\left(a_{t}|s_{t}\right)},1-\epsilon ,1+\epsilon \right){\hat{A}}_{t}\right)\right]$$

where π and $\pi_{old}$ are the current and previous policies respectively, ${\hat{A}}_{t}$ is the advantage function and ϵ is a hyperparameter to clip the value function. For more details about PPO, the readers are referred to [30].

#### 3.2.4. Reward Shaping

For the reinforcement learning problems, the training performance is highly related to the reward function. We use a reward shaping function including the following three parts:

(5) $$r=-w_{ctrl}*r_{ctrl}+w_{dist}*r_{dist}+w_{grasp}*r_{grasp},$$

where $r_{ctrl}$ is the action control reward, $r_{dist}$ is a dense reward decided by the distance between gripper and object, $r_{grasp}$ is a sparse reward for a successful grasping, and *w* are the corresponding weights balancing the rewards. In particular, $r_{ctrl}$ is introduced to smooth the control action and it is formulated as

(6) $$r_{ctrl}=\sum_{i}a_{i}^{2},$$

where $a_{i}$ is the action control signal. From Section 3.2.2 we can know that the action includes $a_{arm}\left(\delta x,\delta y,\delta z\right)$, $a_{base}\left(\delta x,\delta \theta \right)$ and $a_{grip}$. $r_{dist}$ is defined as the Euclidean distance between the gripper and the target object:

(7) $$r_{dist}=\sqrt{\delta_{x}^{2}+\delta_{y}^{2}+\delta_{z}^{2}},$$

where $\delta_{x},\delta_{y},\delta_{z}$ represent the relative position between object and gripper in x,y,z directions, respectively. The grasping reward $r_{grasp}$ is a large reward when the gripper picks the object successfully.

#### 3.2.5. Deep Reinforcement Learning Control

**Figure 2:**
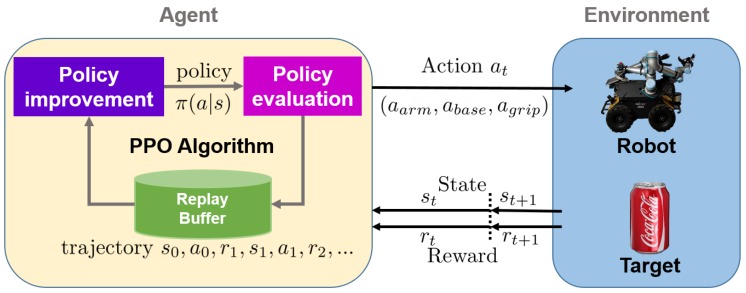
Deep reinforcement learning control and policy training. The action $a_{t}$ includes arm action $a_{arm}$, robot base action $a_{base}$ and gripper action $a_{grip}$. The PPO algorithm samples the state-action from the replay buffer for training and updating the policy π(a|s) after some timesteps.

Based on the above introduction, the whole deep reinforcement learning control process is given in Figure 2.

### 3.3. Visual Perception

Mobile manipulation usually requires 6-DoF object poses for planning and grasping. Therefore, it is necessary to have a visual perception system estimating object poses. In order to enable efficient training and better generalizability, we obtain the objects’ poses directly through the simulator when training the deep reinforcement learning policy, without relying on an end-to-end training. Then, the trained models are flexible to benefit from any object pose estimation technique when being deployed in reality. In this work, we choose Nvidia’s Deep Object Pose Estimation (DOPE) [33] as the vision algorithm to estimate the 6-DoF object pose from a single RGB image of an on-board camera. Note that different from the robot grasping using static arms, the on-board camera is essential for mobile manipulation in unstructured, dynamic environments.

**Figure 3:**
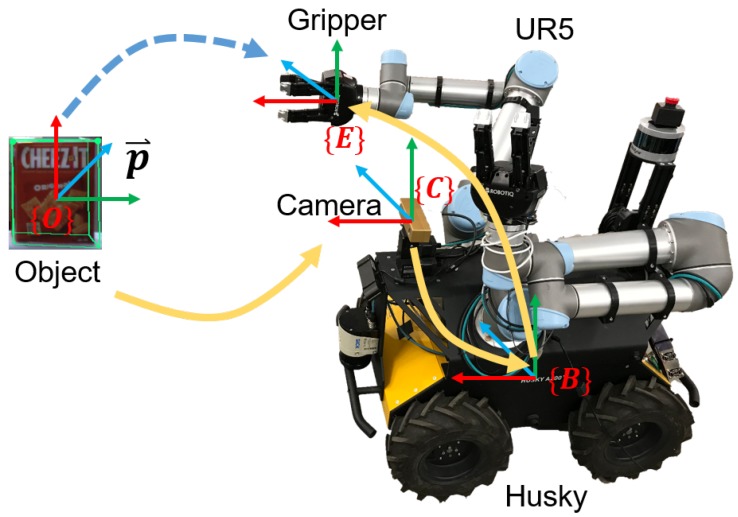
Coordinate frames transforming from the camera frame to the robot and gripper frames.

After the object pose is estimated from the vision model, it is transformed from the camera coordinate frame to the robot local base frame for grasping. The system transformation is shown in Figure 3. Then, the object position is represented w.r.t robot base frame as one of the state inputs of the deep reinforcement learning.

## 4. Simulation

This section focuses on the simulation evaluation of the proposed mobile manipulation algorithm based on deep reinforcement learning. The simulation environment setting is first introduced, followed by the definition of the mobile manipulation task and the simulation evaluation.

### 4.1. Environment Setting

**Figure 4:**
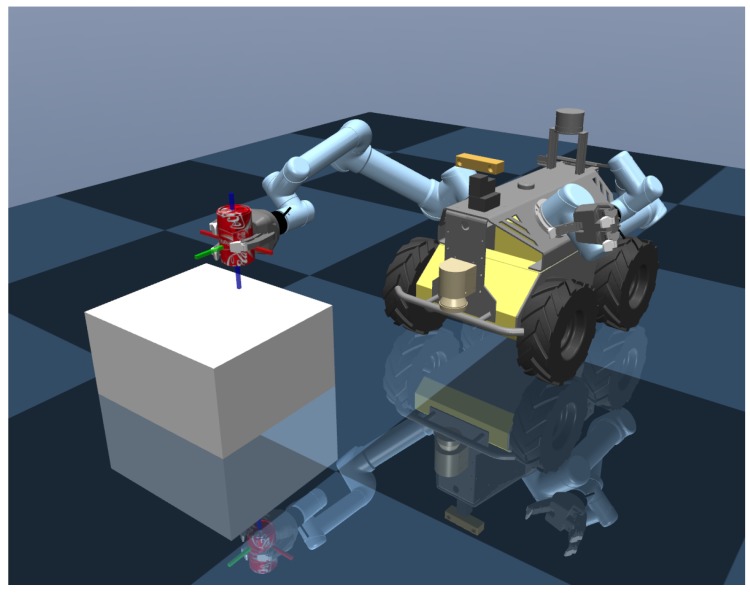
Husky Dual UR5 MuJoCo Environment we built for a basic mobile manipulation task.

First, a new OpenAI Gym [34] MuJoCo simulation environment is created for simulating the mobile manipulation scenario and training the policy. Since we will use the dual-arm Clearpath Husky mobile robot in practice in Section 5, the simulation is designed by using an official Husky Dual UR5 robot Universal Robotic Description Format (URDF) model with two UR5 manipulators plus Robotiq 3 finger grippers. The simulation environment we built is shown in Figure 4.

#### Task Definition

We choose a classical mobile manipulation task, i.e., mobile picking task, for the evaluation of the proposed mobile manipulation system. The task aims to autonomously recognize and pick a random object on a desk, starting from any initial position which is out of the arm’s workspace. This mobile manipulation task is challenging because it requires a feasible (if not optimal) policy which seamlessly considers both the locomotion and manipulation, based on the on-board sensors. Then, the task pipeline is following:

- detecting the target object and estimating its 6-DoF pose using an on-board RGB camera;
- controlling the robot and the arms to approach the target without collision;
- picking the object up using the policy generated by deep reinforcement learning.

**Figure 5:**
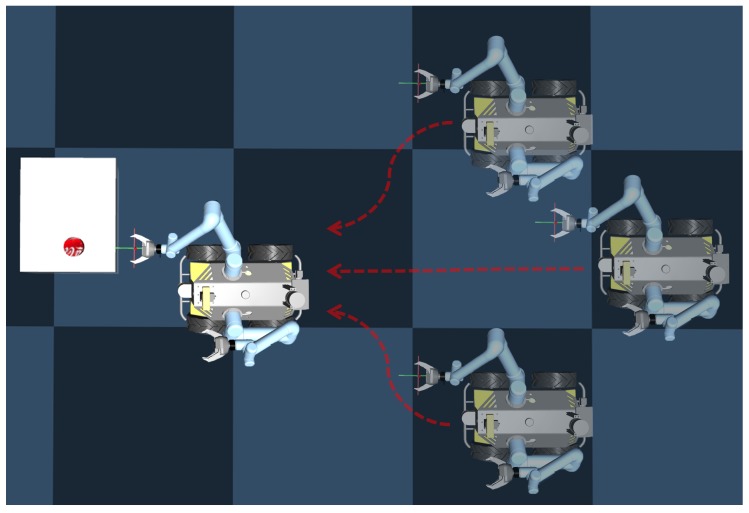
Husky robot mobile picking task from three random initial positions.

The whole picking-up progress is shown in Figure 5.

### 4.2. Training Process

To speed up the training process, we use the distributed reinforcement learning library, RLlib [35]. RLlib is an open-source library for reinforcement leaning that offers high scalability algorithms for a variety of applications. Rllib is based on ray [36], a fast and simple framework for building and running distributed applications. The distributed model selection tool, Tune [37], is also utilized for hyperparameter tuning. The hyperparameters used in PPO algorithm are listed in Table 1.

### 4.3. Simulation Results

#### 4.3.1. Proposed Mobile Manipulation System

**Figure 6:**
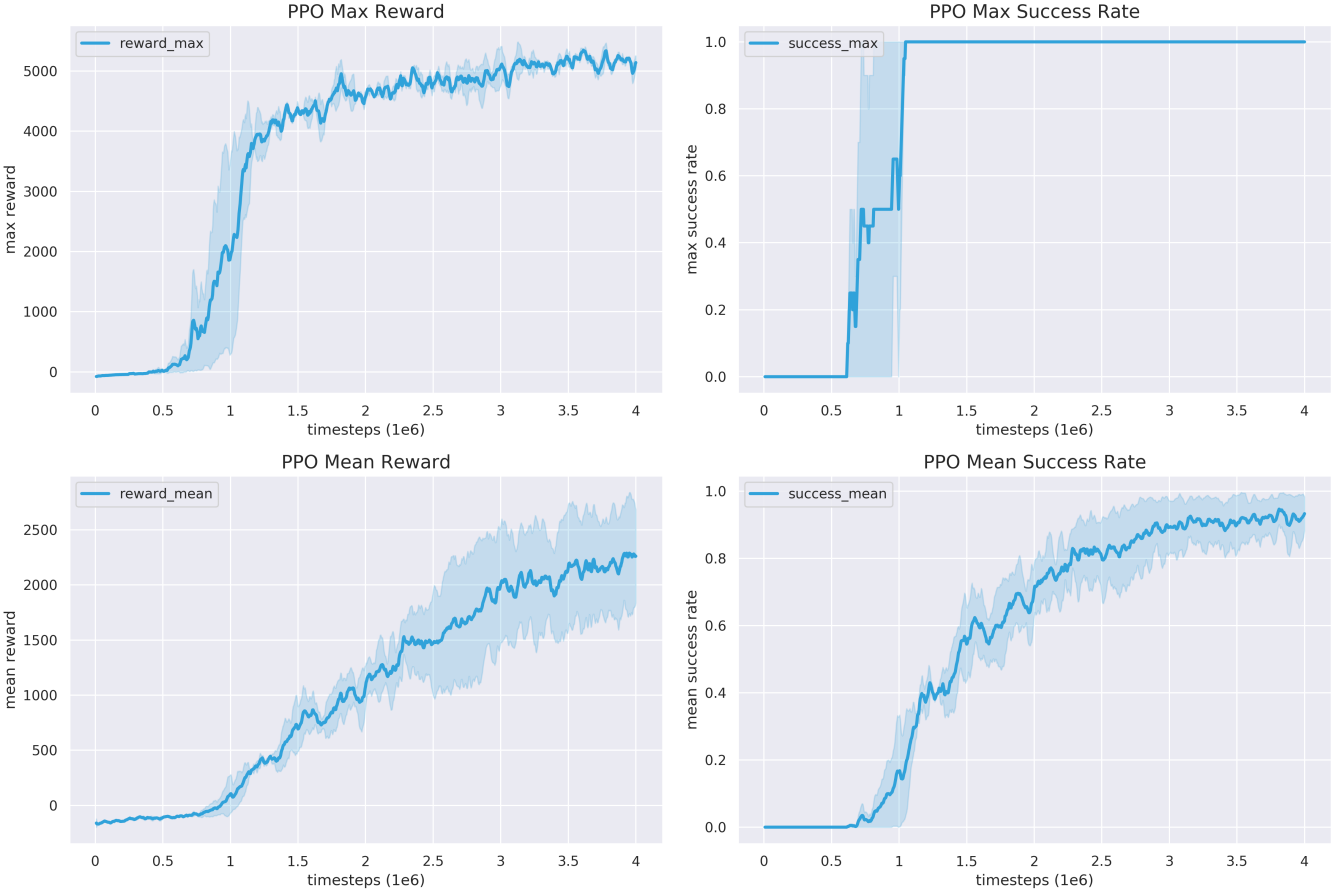
PPO training results. We choose 3 different seeds to train with total 4M episodes. The maximum reward and success rate converge fast to their maxima although their mean values grow relatively slow.

The training results of the proposed mobile manipulation system is given in Figure 6, including the maximum and mean rewards and success rates. It can be seen that at the early stage of the training process, the reward grows very slowly and the success rate is close to 0. However, from 0.5 M to 1.5 M steps, both the reward and success rage have a very sharp increase. When testing the trained model, the robot sometimes is able to pick the object up. But the success rate is not stable. After 3M step, the training converges with reasonable reward and success rate.

**Figure 7:**
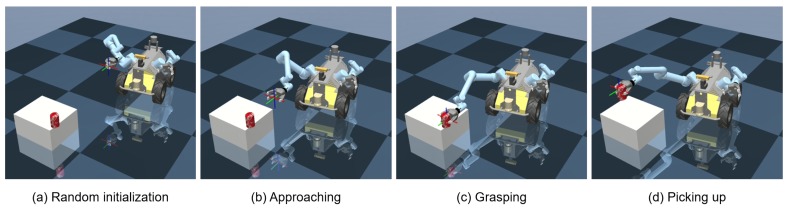
A test case of the proposed mobile manipulation system: (**a**) The positions of the robot and the object are randomly initialized; (**b**) The robot and manipulator approach to the object by using the reinforcement learning policy generated; (**c**) When closing to the object, the gripper grasps it; (**d**) Finally, the object is picked up.

After the training, the mobile manipulation model is tested. Figure 7 shows one of the testing cases. First, the positions of the robot and the target object (a can in this case) are randomly initialized in the environment. Then, based on the object position and the robot state, the mobile manipulation policy is produced by the proposed deep reinforcement learning framework, driving the mobile base to approach the object. Meanwhile, the actions for the manipulation are generated for the manipulation control and grasping. When reaching a suitable position, the gripper picks up the target object, successfully finishing the task.

#### 4.3.2. Comparison with the State-of-the-Art

**Figure 8:**
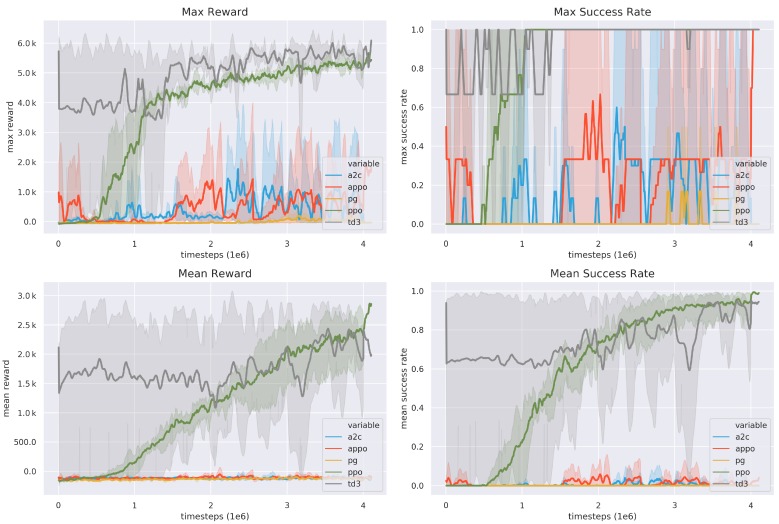
The compare with different RL algorithms. Each algorithm chooses 3 different seeds to train with a total of 4M episode. The PPO algorithm tends to be more stable than others. Note that A2C, APPO, PG cannot fulfil this task. TD3 can achieve the task but the training result is not very stable and have a large variance.

To further evaluate the proposed system, several state-of-the-art RL algorithms are compared for the mobile manipulation task, including APPO [30], TD3 [39], A2C [40], PG [27] and PPO. The reward and success rate are shown in Figure 8. It can be seen that the variance of the proposed method based on PPO is much smaller than the others’, achieving a stable performance. Therefore, the PPO based system is selected for the mobile manipulation in practice.

#### 4.3.3. Different Scenarios

**Figure 9:**
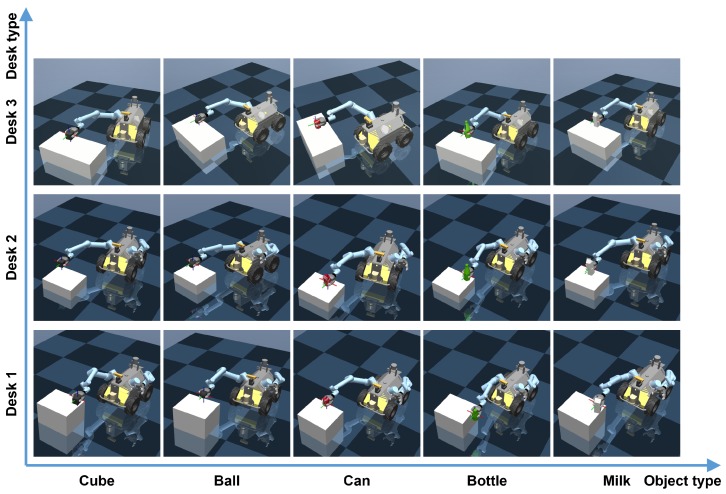
Testing in different scenarios with 5 different types of objects (cube, ball, can, bottle, and milk carton). Desk 1 with a height of 0.20 m; Desk 2 with a height of 0.1 m; Desk 3 with a height of 0.15 m.

In this part, we evaluate our model performance in different scenario configurations on the desk type and target object type. We choose three different desks and five different objects, including a cube, a ball, a can, a bottle and a milk carton. Desk 1, 2 and 3 are desks with a height of 0.20 m, 0.10 m and 0.15 m, respectively. For the different objects, the ball has a sphere shape and tends to be hard to grasp, the milk carton has a slim cube shape, the bottle has a slim cylinder shape, and the cube is a simple basic shape. These objects well represent most of the common objects a robot arm may need to interact with in practice. Figure 9 presents the results of these tests. It can be seen that the mobile grasping system proposed has a good performance and robustness and can also generalize to some new situations which are not trained specifically.

Table 2 summarizes the success rates in different situations with various objects. From the results, it can be seen that the system achieves high success pick-up rates except for the ball whose sphere shape could lead to unstable grasping. In some grasping cases, the ball may drop off from the gripper. Meanwhile, since the policy is trained using the middle height desk, the two success rates of using low and high desks are slightly lower.

## 5. Real Experiment

**Figure 10:**
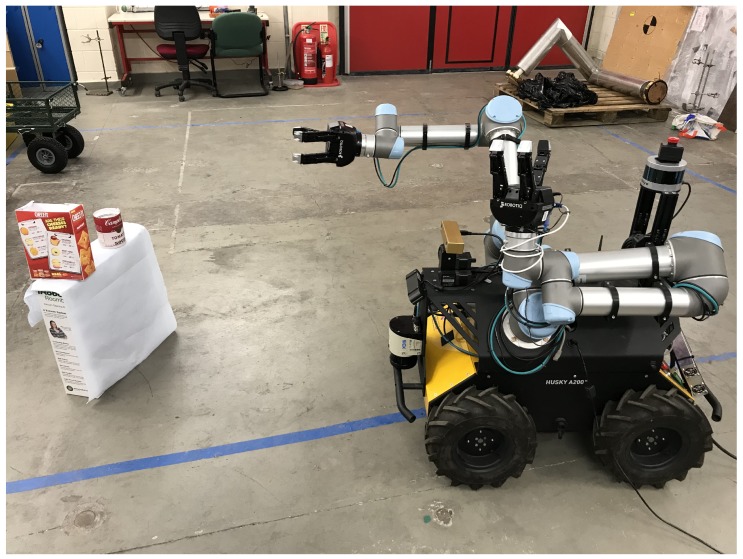
Clearpath dual UR5 arm Husky robot used for the real-world experiments.

To evaluate the learned policy in practice, we test the trained model and policy in the real environment. As shown in Figure 10, a Clearpath dual UR5 arm Husky robot is used to perform the mobile manipulation task, based on an on-board RGB camera.

### 5.1. ROS-Based Control Framework

**Figure 11:**
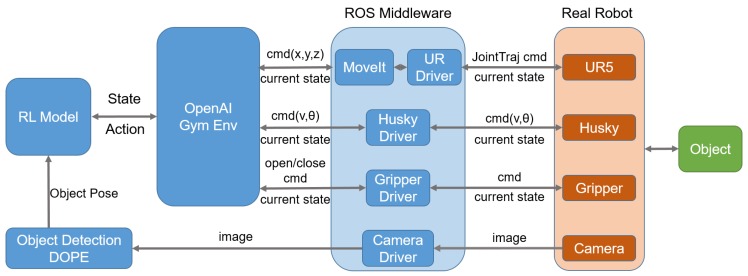
ROS-based control framework.

For the real robot, we use a Robot Operated System (ROS) [41] based control system to apply the trained policy on the mobile base and the manipulators. The ROS framework is presented in Figure 11. The ROS middleware includes the drivers of the UR5 arms, Husky mobile base, gripper and the camera. Both the state and control of the robot are realized in ROS as well.

### 5.2. Hand-Eye Calibration of Arm and Camera

**Figure 12:**
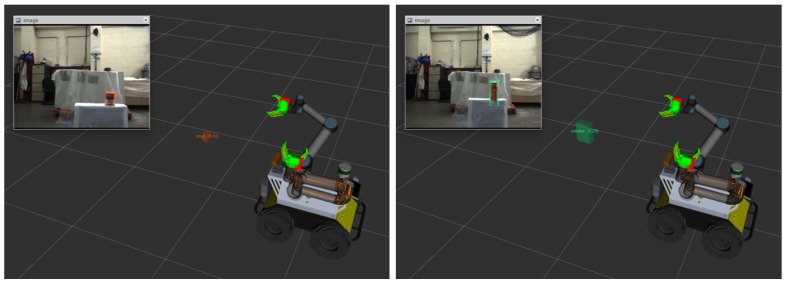
Grasping different objects in RViz visualization.

As discussed in Section 3.3, the coordination transformation between the arm and the camera needs to be available in order to obtain the poses of the object in the arm’s coordination frame. Therefore, a eye-on-hand configuration is employed. The arm-camera calibration method can be found in [42]. The visual perception system estimates the pose of an object and then transforms it from the camera frame to the arm frame in real-time. Some examples in the ROS RViz visualization are given in Figure 12, describing the relative position between an object and the robot.

### 5.3. Experimental Results

**Figure 13:**
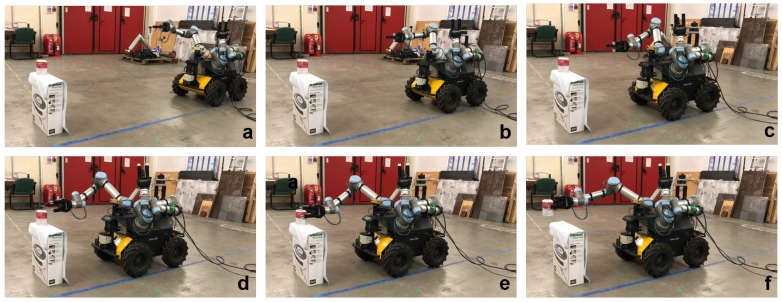
Real mobile grasping process for a soup can. (**a**) is starting, (**b**–**d**) is approaching, (**e**) is grasping, (**f**) is picking up.

**Figure 14:**
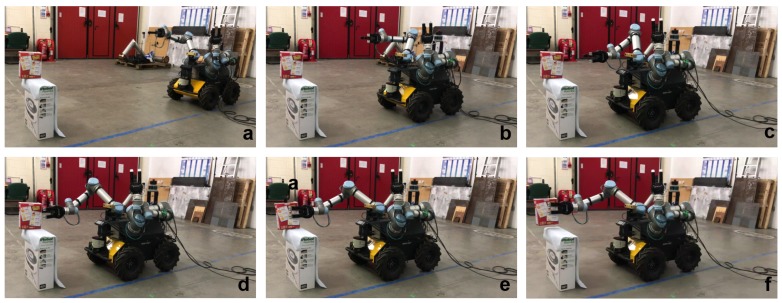
Real mobile grasping process for a cereal box. (**a**) is starting, (**b**–**d**) is approaching, (**e**) is grasping, (**f**) is picking up.

The trained model and policy are evaluated to grasp two different objects, i.e., a soup can and a cereal box, in reality. For the real experiment, the positions of the robot and the UR5 arms are randomly initialized. The vision system detects the object of interest and estimates the relative position between robot and the object. Once a relative pose is estimated, the robot is controlled to move towards the object with the arm motion planning generated by the deep reinforcement learning policy. When the gripper is positioned close to the object, its fingers are given the action from the policy. Some of the real robot grasping examples are shown in Figure 13 and Figure 14.

The real experiments verify that the proposed mobile manipulation system can achieve last-stage mobile grasping autonomously although mobile manipulation in unstructured environments is recognized to be challenging, considering the complexity of the mobile base, arm, gripper and vision subsystems. It is appealing that a deep reinforcement learning policy trained in simulation can be successfully generalized to the real robot. This is because the proposed system decouples the visual perception system from the end-to-end deep reinforcement learning training process. However, there are some differences between the simulation and the real experiment. In the simulation, the mobile base can keep moving or halt at a suitable position while the arm reaches and grasps the object. In the real experiment, the motions of the arm and mobile base are decoupled since the on-board camera used for object detection may not be able to observe the object of interest (due to its limited field of view and occlusion from the mobile manipulator) when they are too close. Therefore, once the mobile robot base reaches a position where the object is within the arm’s operating area, it stops its movement before the arm continue to move. We will investigate how to closely couple the mobile base and arm in practice in our future work.

It is worth noting that the success rate of the real robot degrades in practice compared with the simulation because of the differences between the simulation and the real world on the robot dynamics, object pose estimates, environment backgrounds, etc. After the in-depth analysis, it is believed that the failure of object detection caused by the occlusion from the mobile manipulator is also one of the main reasons for the low success rate in practice. In our future work, we will add an additional camera on the wrist of the arm to tackle this problem.

## 6. Conclusions

In this paper, we propose a learning based mobile manipulation system using PPO algorithm. The mobile manipulator is a complex system with an arm, a mobile base, a gripper and a vision system. We use a whole-body learning policy to control the robot and train the policy in a simulator firstly. Given the learned policy, our mobile manipulator can achieve mobile picking task autonomously only based on the on-board sensors. The learned policy is tested with different objects in various scenarios. Thanks to the design of the mobile manipulation system, the policy trained in the simulation can be successfully transferred to real robot without fine-tuning.

The future work will focus on improving the real-world performance and robustness. First, we will study how an extra camera on the arm wrist would increase the success rate of the real tests. Second, we would like to extend the learnt policy to more complex tasks, such as having obstacle avoidance and autonomous navigation capability in a larger open area.

## Figures and Tables

**Table 1:** Hyperparameters used for PPO.

Hyperparameter	Value
hardware configuration	3 NVIDIA GPUs + 32 CPU cores
discount factor γ	0.99
Generalized Advantages Estimation λ	0.95
PPO clipping parameter ϵ	0.3
optimizer	Adam [38]
learning rate	0.00005
sample batch	200

**Table 2:** Mobile Manipulation success rate in different situations (total = 20).

Object Type	Desk Height		
	Low	Middle	High
ball	0.5	0.7	0.6
milk carton	0.6	0.8	0.7
cube	0.8	0.9	0.9
bottle	0.8	0.9	0.7
coke can	0.7	0.8	0.8
